# Protocol for the development of a Core Outcome Set (COS) for Adolescents and Young Adults (AYAs) with cancer

**DOI:** 10.1186/s12885-023-11716-2

**Published:** 2024-01-24

**Authors:** Olga Husson, Silvie H. M. Janssen, Bryce B. Reeve, Samantha C. Sodergren, Christabel K. Cheung, Martin G. McCabe, John M. Salsman, Winette T. A. van der Graaf, Anne-Sophie Darlington

**Affiliations:** 1 Department of Medical Oncology, Netherlands Cancer Institute, Amsterdam, The Netherlands; 2 Department of Surgical Oncology, Erasmus MC Cancer Institute, Rotterdam, The Netherlands; 3 Division of Psychosocial Research and Epidemiology, Netherlands Cancer Institute, Amsterdam, The Netherlands; 4 Department of Population Health Sciences, Duke University School of Medicine, Durham, NC USA; 5 Duke Cancer Institute, Durham, NC USA; 6 School of Health Sciences, University of Southampton, Southampton, UK; 7 School of Social Work, University of Maryland, Baltimore, MD USA; 8 Division of Cancer Sciences, University of Manchester, Manchester, UK; 9 The Christie NHS Foundation Trust, Manchester, UK; 10 Department of Social Sciences & Health Policy, Wake Forest School of Medicine, Wake Forest Baptist Comprehensive Cancer Center, Winston Salem, NC USA; 11 Department of Medical Oncology, Erasmus MC Cancer Institute, Erasmus University Medical Center, Rotterdam, The Netherlands

**Keywords:** Adolescents and young adults, AYAs, Oncology, Core outcome set, Core measurement set, COMET, Outcome, Consensus

## Abstract

**Background:**

Adolescents and young adults (AYAs) with cancer, defined as individuals aged 15–39 years at initial cancer diagnosis, form a unique population; they face age-specific issues as they transition to adulthood. This paper presents the protocol for the development of a core outcome set (COS) for AYAs with cancer.

**Methods:**

The methodological standards from the Core Outcome Measures in Effectiveness Trials (COMET) and the International Consortium for Health Outcomes Measurement (ICHOM) for COS development will guide the development of the COS for AYAs with cancer. The project will consist of the following phases: (1) define the scope of the COS; (2) establish the need for a COS in this field (3) assemble an international, multi-stakeholder working group; (4) develop a detailed protocol; (5) determine “what to measure” (i.e., outcomes); (6) determine “how to measure” (i.e., measures); and (7) determine “case-mix” variables.

**Conclusions:**

The development of a COS for AYAs with cancer will facilitate the implementation of efficient and relevant standards for data collection, both for clinical trials and in routine healthcare, thereby increasing the usefulness of these data to improve the value of the care given to these underserved young cancer patients.

**Supplementary Information:**

The online version contains supplementary material available at 10.1186/s12885-023-11716-2.

## Introduction

Cancer patients in any healthcare system should have access to high-quality care to ensure good outcomes in terms of survival and health-related quality of life (HRQoL) [1, 2]. This is particularly important for adolescents and young adults with cancer (AYAs), defined here as those aged 15 to 39 years at initial cancer diagnosis [3]. AYAs’ annual cancer incidence is 42.2/100.000, with 1,231,007 cases worldwide reported in 2018 (together 6.8% of all cancers) [4]. Advances in cancer treatment have led to increased survival rates for AYAs combined with over 85% surviving at least five years in the developed world [5]. Despite the relatively favourable cancer prognosis, AYAs are at risk of treatment-related medical effects (e.g. cardiovascular disease or second malignancies), infertility, psychosocial effects (e.g. difficulty in romantic relationships) and financial instability (due to unemployment without a prior career), and have an increased risk of late mortality [6–8].

AYAs are distinct from younger and older cancer populations based on their unique spectrum of cancer types, an evolving physiological milieu characterised particularly by endocrine and neurodevelopmental maturation, and in some cases characteristic genomic alterations that vary with age, resulting in differences in the biological and clinical behaviour of their tumours [3, 9–11]. AYAs experience difficulties in accessing specialised care and diagnostic delays due to a lack of awareness among both patients and healthcare professionals that cancer may occur in this age group [12]. A scarcity of AYAs’ participate in clinical studies (ranges from 5 to 34%) limiting the evidence base for treatments [13]. For some tumour types (brain tumours and sarcomas), survival in AYAs is poorer than in children with the same diseases [14]. In addition, a cancer diagnosis challenges AYAs’ abilities to achieve a multiplicity of developmental milestones that are characteristic for their age and heterogeneity of social and cultural contexts [15–17]. Examples of these milestones include forming one’s own identity and a healthy body image, establishing autonomy, responsibility, and independence, finishing education, and starting a career, starting a romantic relationship, and having and raising young children. AYAs’ cancer experiences have life-long impacts on their physical, emotional, cognitive, and social quality of life [18]. Equally, AYAs also report positive consequences of having had cancer such as improvements in family relationships, greater maturity, changes in priorities, a focus on strengths, and reflections on life purpose [16, 19, 20].

AYAs require access to oncology services that provide expert cancer care and consider their age-specific and complex psychosocial and physical needs. In many parts of the world, AYAs face inequalities of care as they are poorly served by the traditional dichotomy of the integrated paediatric (“patient/family-centered”) care services versus dispersed (“disease-centered”) adult oncology services [13, 21, 22]. Up to half of AYAs report unmet service and informational needs, impacting their direct (survival rates) and indirect (long-term effects and mental health) recovery and return to participation in society [23].

A European AYA focused Working Group (WG) has highlighted the need to improve health outcomes for AYAs [13], with emphasis on AYA-specific needs. They report an absence of outcome measures to monitor and evaluate AYA care programs. Findings and recommendations of the AYA WG are in line with other reports, like the U.S. National Cancer Institute’s ‘Closing the Gap: Research and Care Imperatives for Adolescents and Young Adults with Cancer’ [3].

There is a clear need to comprehensively assess AYAs’ specific needs, including screening for physical and psychosocial problems and providing multidisciplinary, holistic, age-specific hospital and community support. This includes fertility counselling, psychological support (e.g., body image, sexuality, and relationships), and occupational and financial support services (e.g., education and career development). Although an ever-increasing amount of data is available, the collection, access, and use of these data are still very fragmented within and especially across national healthcare systems. There is a lack of international data standardization, data interoperability and prospective collection of outcomes of relevance for AYAs that are important for informing decision-making. Thus, the field needs a core outcome set (COS), derived from a multi-stakeholder consensus-based process, that includes the minimum set of AYA-specific outcomes and associated high quality measures of those outcomes. An AYA-specific COS will enable data-driven healthcare innovation and serve as a basis to address clinically relevant questions in providing inclusive care that responds to needs of the whole person for this vulnerable patient group [24–26]. We aim to describe the protocol to develop a consensus-based COS for AYAs for future use in research and clinical practice.

## Materials and methods

**Fig. 1:**
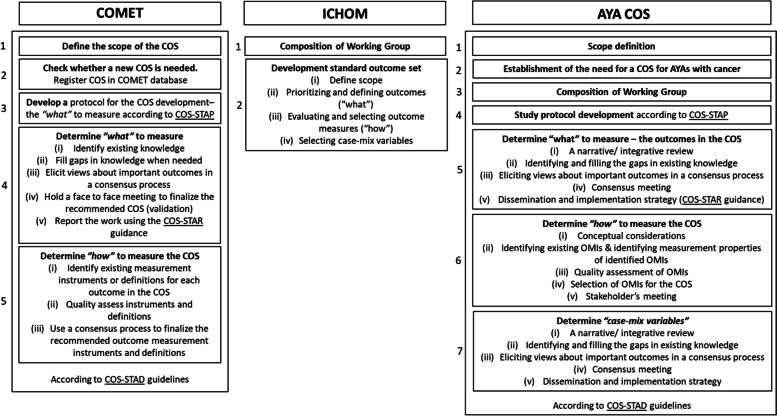
Seven steps of the COS development

Our study design was informed by existing methodological standards of the Core Outcome Measures in Effectiveness Trials (COMET) initiative [27], and the International Consortium for Health Outcomes Measurement (ICHOM) [28]. We will use an adapted, combined version that captures all methodological standards of both COMET and ICHOM as a guideline for this protocol, resulting in the following phases: 1) scope definition, 2) establish the need for a COS, 3) assemble a working group, 4) develop a study protocol, 5) establish what to measure, 6) determine how to measure outcomes, and 7) identify case-mix variables (Fig. 1). These phases are outlined in more detail below; we have published an opinion paper identifying a clear need for and defining the scope of a COS for AYAs [2].

In developing the COS for AYAs, we will adhere to the 11 minimum standards of Core Outcome Set-STAndards for Development (COS-STAD) [29]. In addition, the protocol for the COS development will be developed according to the 13 items considered essential documentation in a protocol as described in the Core Outcome Set-STAndardised Protocol Items (COS-STAP) [30]. Furthermore, in reporting on the COS for AYAs, we will adhere to the Core Outcome Set STAndards for Reporting (COS-STAR) Statement as a reporting guideline for COS studies [31].

### Phase 1: scope definition

The scope in terms of health condition, target population, intervention and setting are described in Table 1.

**Table 1:** Developing the COS process based on the COMET COS-STAD

Domain	Standard number	Methodology	Application in the proposed project
Scope specification	1	The research or practice setting for the COS	Research and clinical practice
	2	The health condition covered by the COS	Cancer
	3	The population covered by the COS	AYAs aged 13–39 years at initial cancer diagnosis and inclusive of the whole cancer care continuum
	4	The interventions covered by the COS	All
Stakeholders involved	5	Those who will use the COS in research	Researchers, policy-makers dealing with AYAs
	6	Healthcare professionals with experience of patients with the condition	Clinicians (e.g. medical oncologists), nurse specialists, applied healthcare professionals
	7	Patients with the condition or their representatives	Adolescents and young adults aged 13–39 years at first cancer diagnosis, their informal caregivers or advocates
Consensus process	8	Long-list of outcomes considered by stakeholders	Literature review and interviews with stakeholders
	9	Scoring process and consensus definition	Delphi scoring using a nine-point Likert scale (1–3, limited importance; 4–6, not crucial importance; 7–9, crucial importance) Consensus criteria: score of 7 + per item by ≥ 70% of respondents
	10	Criteria for including/eliminating outcomes	Inclusion: outcomes scored ‘critically important’ by ≥ 70% AND ‘not important’ by < 15% of participants Exclusion: outcomes scored ‘critically important’ from less than 50% of participants
	11	Avoiding language ambiguity in the description of outcomes	Plain language version will be available, informed by interviews and pilot-tested with patients

### Phase 2: establishment of the need for a COS for AYAs

According to the COMET database, there is no COS available or being developed for AYAs (July 15 2022).

### Phase 3: composition of AYA Working Group

The Working Group consists of a steering committee and a project advisory group (PAG). The steering committee is responsible for the day-to-day management of the project. It consists of two epidemiologists (OH, BBR), three psychologists (SS, JMS, ASD), one social researcher (CKC), two clinicians (MGM, WTAG) and one patient representative (CKC). The PAG consists of international experts (AYAs and their caregivers/representatives, health care practitioners from different disciplines, researchers, regulators, policy makers and all other important decision-makers) who will provide their input at critical points of the study such as protocol development, stakeholder recruitment and the consensus meeting. We will make sure that the PAG members represent the heterogeneity of the AYA cancer population in terms of clinical (e.g., tumour type) and demographic characteristics (e.g., age group, gender), healthcare systems and countries. Age groups will be represented in three groups, including adolescents (13–17 years old), emerging adults (18–25 years old) and young adults (26–39 years old). We decided to use 13 years instead of 15 years as cut-off to be as inclusive as possible, given that in some healthcare systems lower age ranges are used to define AYAs.

### Phase 4: study protocol development

This COS development protocol was created according to the 13 items considered essential documentation as described in the COS-STAP [30] and the 11 minimum standards for COS-STAD – Table 1) [29].

### Phase 5: determine “what” to measure – the outcomes in the COS

Existing knowledge on potentially relevant outcomes needs to be identified to inform the consensus process. Two data sources will be used in our project: step 1) Literature review of published studies; step 2) Interviews and focus groups with key stakeholders.

#### Step 1: literature review

No similar or ongoing systematic reviews are recorded in the international prospective register of systematic reviews (PROSPERO). Therefore, a computerized, structured literature search of different databases (Medline ALL, Embase, Web of Science Core Collection, Cochrane Central Register of Controlled Trials, and additional search engines (Google Scholar)) will be performed to identify relevant outcomes for AYAs. The search string for each database has been constructed together with an experienced librarian and can be found in Additional file 1: Appendix Text A1. The protocol for this review is registered on OSF Registries (https://osf.io/vg2a3).

Inclusion criteria:

- Population: AYAs (13 up until (and including) 39 years old at initial cancer diagnosis) or a subset of this age range (e.g. adolescents or young adults only). The AYA age range will be flexibly applied, meaning not limited to 13–39 years, because lower and upper age limits for AYAs differ per country or per study
- Mixed samples will be included if age stratified outcomes are available for the target population
- Studies conducted in other study populations, such as healthcare providers, friends, parents or carers of AYAs will be included only if the participants provide information on the outcomes of AYAs
- On and/or off treatment, including at diagnosis, during treatment or following treatment (patients and/or survivors). There is no upper limit for the time since diagnosis for AYA cancer survivors. Patients on maintenance treatment will be included.
- Written in English language
- Any type of malignant tumour or benign central nervous system (CNS) tumour
- Study types: randomized controlled trials (RCTs), observational cohort studies, case–control studies, case series, cross-sectional studies, qualitative studies
- Studies focussing on all types of biological, physical, psychological or social outcomes

Exclusion criteria:

- Study population consists exclusively of healthy or non-cancer study population, including desmoids and in situ carcinomas
- Study population consists exclusively of childhood cancer patients aged under 13 years at initial cancer diagnosis or adult cancer patients aged over 39 years at initial cancer diagnosis
- Full text is unavailable
- Only a conference abstract or poster is available
- Study protocols, case reports, reviews/ meta-analyses, expert opinions, theoretical papers, policy documents/ guidelines, consensus letters, editorials
- Non-human study

Each article will be independently screened by two reviewers based on title/abstract and full text, using the selection criteria outlined above. Conflicts will be resolved through discussion or, if necessary, by involving a third reviewer from the steering committee to arrive at majority agreement.

The following data will be extracted: study characteristics, outcomes, outcome measurement instruments and/or definitions provided by the authors for each outcome as described in the COMET handbook [27] and potential case-mix variables (e.g. sex, ethnicity, type of treatment) as preliminary work for Phase 7—determine “case-mix “ factors.

All outcomes will be extracted verbatim from the source manuscripts to allow external critical review of the COS right back to its inception. It is likely that some outcomes will be the same but will have been defined or measured in different publications in various ways. For example, literature on work or employment outcomes often uses different definitions e.g., impact of cancer on work plans [32], vocational goal disruption [33], return to work rates [34], workability and employment status [35]. The first step is to group these different definitions together (extracting the wording description verbatim) under the same outcome name. Subsequently, these outcomes will be grouped into outcome domains. A generic taxonomy has been developed for outcomes in medical research, containing 38 outcomes, which will be used as a framework for our project [36], including medical outcomes (e.g., cardiac, endocrine), life impact outcomes (e.g. social functioning, global quality of life) resource use (e.g. economic, hospital) and adverse treatment events. Categorisation of each verbatim outcome definition to an outcome name and each outcome name to an outcome domain will be performed independently by two reviewers from multidisciplinary backgrounds. Where two researchers work on this process, another steering committee member will need to resolve differences and make final decisions in alignment with the SC’s vision.

At a later stage we will also categorize whether an outcome is age-specific or cancer-generic/tumour-specific and at which phase of the cancer continuum the outcome is of importance (all phases, diagnosis, treatment, follow-up, survivorship, palliative care and end of life; Fig. 1). This will be done by the same reviewers.

#### Step 2: interviews and focus groups with stakeholders

Interviews and small group interviews with approximately 30 AYAs and others stakeholders (up to 20 carers and up to 20 health care professionals) will be carried out to be able to generate a complete list of outcomes of relevance to AYAs.

##### Stakeholders (AYAs, caregivers and HCPs)

We will use purposive (aiming for diversity), rather than convenience sampling. To ensure a broad range of views are represented, we will recruit participants through clinical settings and online communities. This will assist recruiting participants from multiple countries and settings, and with different backgrounds and experiences. More specifically, AYAs and caregivers/representatives will be recruited via the treating HCPs who are active in the international AYA oncology community (via ENTYAC, ESMO-SIOP, Global AYA conference [13]) and via diverse international patient advocacy groups (e.g. Youth Cancer Europe, Canteen Australia, Teen Cancer America, Stupid Cancer, Teenage Cancer Trust, Young Lives versus Cancer, SHINE). Caregivers can include partners, parents, siblings and other important caregivers as identified by the AYAs who participate. A stratification matrix will be composed to ensure a diverse AYA cancer population is included in terms of age as the primary category (adolescents, emerging adults, and young adults), and sex, tumour and treatment characteristics, country, racial and ethnic background (Additional file 1: Appendix Table A1). HCPs will be recruited via national and international AYA oncology networks (e.g. ENTYAC, ESMO, SIOP).

##### Data collection

A semi-structured interview guide will be developed to elicit outcomes of importance to AYAs. Interview schedules will be prepared considering similar qualitative studies and results of the literature review. Interview schedules will be prepared in English for each group and pilot-tested by AYAs, caregivers/representatives, HCPs from different disciplines, researchers, regulators, policy makers and all other important decision-makers. Ethical approval will be obtained in advance from the University of Southampton UK. All interviews will be conducted remotely via videoconferencing or telephone and will be audio-recorded.

##### Data analysis

Interviews will be transcribed verbatim by a transcription company, with a steering committee member regularly (5% of transcripts) checking the quality of the transcripts. NVivo qualitative data analysis software will be used. Transcripts will be analysed using thematic analysis methods [37] and will be guided by the generic taxonomy as described above. We will define saturation to have been reached when themes recur and no new information is captured from subsequent interviews.

The information will be used to supplement the list of potential AYA-centred outcomes gathered from the literature or create new outcome domains that are missing from the framework [38, 39]. The Consolidated criteria for Reporting Qualitative research (COREQ) will be used to report the results of the qualitative study [40]. The output of step 1 and 2 will be used to inform step 3.

### Generating list of outcomes

The review of existing literature (step 1) and the qualitative research (step 2) has the potential to result in a long list of outcomes. The first step is to determine the inclusion and wording of outcomes to be considered in the initial round of the consensus exercise. The length of the initial list will be discussed with the steering committee and if needed explicit criteria will be formulated to reduce the size. Thereafter the final list of outcomes will be pilot- or pretested using cognitive or ‘think aloud’ interviews to examine how patients and other stakeholders interpret the draft outcomes which will help to refine the outcome labels and explanations [41, 42].

#### Step 3: Delphi process

The Delphi technique is used for achieving convergence of opinion from all stakeholders (with equal contributions) on the importance of different outcomes in multiple anonymised prioritisation rounds [43]. Responses for each outcome will be summarised and fed back anonymously within the subsequent questionnaire. Participants will be able to consider the views of others before re-rating each item and can, therefore, change their initial responses based on the feedback from the previous rounds.

Our Delphi study will consist of three rounds conducted using an online platform to maximise participation and enhance credibility [44].

##### Participants

Three stakeholder group panels will be created: patients and carer/representatives; HCPs; researchers and policy makers. People will only be allowed to participate in one panel. We aim to recruit at least 50 people per stakeholder group. Having different group sizes may downgrade the voice of the smallest group, hence, data analysis will be performed for the different stakeholder groups separately, this will allow for intra- and inter-group variability to be explored and for equal group’s representation in the process.

We will recruit through patient networks (such as Youth Cancer Europe, Canteen, Stupid Cancer, Teenage Cancer Trust, Young Lives versus Cancer, SHINE), professional organisations (such as ENTYAC, EORTC), research networks (such as AYA Cancer Alliance 2022, ECOG-ACRIN), other professional organisations and societies, AYA patients’ groups on social media, and contacting key researchers identified in step 2 (qualitative interviews). We will also encourage snowball sampling, asking participants to forward the invitation appropriately. A member of the research team will check the eligibility of respondents.

An invitation email will also contain an electronic link that will allow stakeholders who are willing to participate to register for the survey and provide their consent for completing all three rounds of the online Delphi survey. Registered participants will receive an email containing a link to round 1 of the survey only after they have consented to participate.

##### Procedure

The three-round online Delphi survey will be administered using an online platform distributed via email. Each round will be open for 3 weeks. Participants who have not responded to the survey will be sent periodic reminders. Participants who do not complete a round will not be invited to the next round. As per recommendations outlined by the GRADE group), each round will use the same nine-point Likert scale to rate the importance of the outcome for inclusion in the COS [45]. A rating of ‘limited importance’ (rating of 1–3), ‘not crucial importance’ (rating of 4–6), or ‘crucial importance’ (rating 7–9) will be used [46–48]. Participants will also be given the option of ‘unable to score’ to allow for the fact that some stakeholder group members may not have the level of expertise to score certain outcomes.

* Round 1* In the first round of the Delphi survey, each stakeholder will be asked to provide some demographic information and then rate the importance of each outcome. The initial list may not be entirely exhaustive; therefore, one open-ended question will be included at the end of round 1 to give participants the opportunity to suggest outcomes they feel are important but have not been included in the survey. In case more than two respondents suggest an outcome, it will be added to the second round.

* Round 2* All participants who completed round 1 will be invited to round 2. All outcomes included in round 1 will be carried forward to round 2. For round 2 of the survey, the participants will receive their individual score for each outcome, the aggregated scores of their stakeholder group, as well as the other stakeholder groups from the previous round, to consider when they are completing the survey [49]. Summary statistics, such as a median or mean (if normally distributed) and the percentage scoring above a pre-specified threshold (for example, 7–9 on a 9-point Likert scale), will be accompanied by graphs to enhance visual presentation. We will conduct a pilot study with a small group of participant representatives to ensure that the feedback is understood. Subsequently, based on this feedback, each stakeholder will be asked to rate each outcome again. In addition, participants in round 2 will be invited to consider if they are willing to attend a face-to-face consensus meeting to discuss the final set of outcomes.

* Round 3* Participants who completed round 2 will be invited to the third and final round. Round 3 of the Delphi survey will contain the list of the outcomes that are rated as critical (rated 7–9) by at least 70% of respondents and rated as of limited importance (1–3 on Likert scale) by 15% or less of all respondents in round 2. The idea is that the majority considers an outcome to be important enough to incorporate in the COS, with only a small minority considering it to have little or no importance [45]. In addition, to ensure that patient prioritised outcomes are not overwhelmed by the two other stakeholder groups, any outcome that had an average public score of 7 or more will also be re-proposed for voting in round 3. Each participant will then be asked to rate each outcome for a final time using the same rating scale used in rounds 1 and 2.

##### Data analysis

Consensus levels will be defined as following:

- Included: a score of 7 to 9 from at least 70% of participants and a score of 1 to 3 from less than 15% of participants in all groups. These items will be included in the final COS.
- Excluded: a score of 7 to 9 from less than 50% of participants in all groups. These items will be discarded from the final COS.
- No consensus: items which do not achieve the inclusion or exclusion criteria. These will be taken into the consensus meeting for discussion and final voting.

The rate of missing and incomplete responses will be reported with the results of the Delphi survey. If a participant did not complete all rounds, available responses will be included in analysis. An analysis will be performed separately for each item, incomplete responses will not be discarded, and available items will be included in the analysis. To test if missing data effect representativeness, the first-round participant profiles will be compared to participants who completed all rounds.

#### Step 4: consensus meeting

Following the Delphi survey, a 1-day consensus meeting will be scheduled, if feasible, coinciding with an academic conference of relevance for AYAs, to discuss the results and recommend the final COS outcome domains. A pragmatic representative sample will be invited from participants who expressed interest in the second Delphi round. Participants may attend in person or virtually by video conference. The chair will be independent, and the facilitator will encourage all stakeholders to have equal input during the meeting, adopting a collaborative approach to achieving consensus. Outcomes that achieved no consensus in the Delphi survey will be subjected to discussion and voting. A modified nominal group technique will be used, which is a structured group discussion that involves generating, defining, and ranking items to reach consensus while limiting individual dominance [50]. This method will involve a series of discussing outcomes, nominating most and least important outcomes by each participant, at least two per stakeholder group, anonymous voting on outcomes, discussion of voting results and agreeing final COS. A nine-point Likert scale will be used for voting, and the same consensus criteria of the Delphi survey will be applied. Voting results will be presented by the end of the meeting for final COS agreement.

#### Step 5: dissemination and implementation strategy

A multi-method approach to dissemination will be adopted. The development of this COS for AYAs will be reported according to the COS-STAR (Core Outcome Set-STAndards for Reporting) guidelines [31]. The final COS will be published in an open access journal and will be accompanied by a brief explanatory document with examples of good reporting to facilitate the use of the COS in practice and research. Lay summaries will be included in dissemination documents. After publication, the COS will also be made available through the COMET database.

In addition, we plan to disseminate the COS at national and international conferences; through relevant professional and patient organisations; translated summaries, clinical trial registries, consumer groups in partner countries, international guideline development groups, policy makers, journal editors and funders of research in the area of AYA oncology. A more detailed dissemination and implementation plan will be developed within the STRONG-AYA project [51].

### Phase 6: determine “how” to measure the COS

Once a COS is defined, it is then important to achieve consensus on how these outcomes should be measured, i.e., which outcome measurement instruments (OMIs) should be selected. There is often a lack of consensus with regard to the selection of OMIs, resulting in variety of OMIs used to measure the same outcome (e.g., biomarkers, assessments by HCPs, imaging and laboratory tests, patient-reported outcomes, performance-based tests), causing inconsistencies in reporting and difficulties in comparing and combining findings. In addition, the quality of existing OMIs varies considerably. We will follow the guideline of the COnsensus-based Standards for the selection of health Measurement INstruments (COSMIN) / COMET for the selection of OMIs [52]. The steps are outlined in Table 2 and described below.

**Table 2:** Proposed CMS development process based on COSMIN / COMET recommendations

Recommendation	Proposed action
Step 1. Conceptual considerations	• Target population: adolescents and young adults with cancer (AYAs) • Intervention: all • Outcomes: All outcomes included in the COS developed in previous phase
Step 2. (a) Identify existing outcome measurement instruments & (b) measurement properties	Outcome measurement instruments used in the literature will be identified as part of the literature review in step 1. When specific international recommendations exist for a certain measure, we will follow these. Specific attention will be paid to the language availability of measures
Step 3. Quality assessment of outcome measurement instruments	• Search for available reviews of evidence for the psychometric properties of each instrument • If possible, a review will be undertaken for instruments where no previous reviews are available
Step 4. Selection of outcome measurement instruments for the COS	A table of the evidence of the psychometric properties with the AYA population and quality for each OMI will be prepared
Step 5. Stakeholder’s meeting	Stakeholder’s meeting to approve instruments selection based on the review results

#### Step 1: conceptual considerations

The construct (outcomes and domains) to be measured and the target population are defined in phase 5: Determine “what” to measure – the outcomes in the COS.

#### Step 2a: identifying existing OMIs

Existing OMIs will be extracted in the literature review of phase 5. We will conduct a rapid scoping of the available literature per outcome domain/outcome to be sure we do not miss relevant OMIs [53–55]. Information regarding instruments used in research will be extracted, they will appropriately be tabulated and classified according to relevant outcomes. The literature searches will be performed according to the COSMIN/COMET guidelines [43]. The COSMIN guideline for systematic reviews of OMIs recommends that those searching the literature for all OMIs do not use search terms to cover “type of OMI” because a wide variety of terminology is used (e.g., OMIs are also termed measures, methods, questionnaires, tests, etc.), which could lead to a high risk of missing relevant studies [56]. There is, however, one exception for patient-reported outcome measures (PROMs): for these a comprehensive PROM filter, developed for PubMed by the Patient Reported Outcomes Measurement Group of the University of Oxford, will be used available through the COSMIN website [57].

#### Step 2b: identifying measurement properties of identified OMIs

Other searches will be run to identify any available systematic reviews, which evaluated psychometric properties of identified instruments. The COSMIN Database of systematic reviews of outcome measurement instruments and PubMed Central will be searched for this purpose. If no systematic reviews can be identified for a certain instrument, or if the available reviews are outdated or of poor quality, a search of recent publications addressing psychometrics will be carried out to access up to date validation information. We will extract study population, instrument characteristics (e.g., length response options, recall period), results of the measurement properties assessed, evidence on interpretability and feasibility (e.g., scores description, use of devices, floor and ceiling effects, minimal important change or difference, ease of standardization and calculation, completion time). For PROMs, mode of administration, original language and available translations will additionally be extracted.

#### Step 3: quality assessment of OMIs

Evaluation of the quality of the measurement properties for each OMI will be assessed using the COSMIN tool, which was developed by COSMIN and COMET [58]. This tool is recommended to evaluate the psychometric properties of patient reported, clinician-reported, and performance-based outcome measurement instrument, in addition to laboratory values. Rated measurement properties include different forms of validity (content, structural, criterion, cross cultural), internal consistency, reliability, measurement error, hypotheses testing, responsiveness, and validation among AYAs. Each measurement property will be rated as positive, intermediate, or negative. Then overall quality of OMI will be rated from high to unknown according to the recommended criteria.

#### Step 4: selection of OMIs for the COS

A table of the psychometric properties and quality for each OMI will be prepared. Criteria will include evidence of validity (e.g. structural, content, criterion, construct, cross-cultural), reliability (e.g. internal consistency, test–retest reliability), responsiveness, and interpretability of scores. Each criterion will be rated as very good, adequate, inadequate, or not reported. For each outcome, OMIs will be recommended based on their quality assessment. An OMI will be recommended only if it meets the following minimum COS inclusion requirements: at least high-quality evidence of good content validity, high quality evidence of internal consistency, test–retest or inter-rater reliability (if applicable), and if it seems feasible. Where there are missing data regarding psychometric properties for an OMI, we will recommend further validation work. These will be added to the final list as provisional outcome measurement instruments.

#### Step 5: stakeholder’s meeting

Finally, we will organize a stakeholder meeting to ensure transparency of the process and approve the final core measurement set. We will invite AYAs, caregivers/representatives, HCPs, researchers, regulators and policy makers to attend. Participants will discuss results from the reviews and recommendations for the final list of instruments and appropriate time points for measurement. A single OMI will be chosen for each outcome, this will be based on discussion of feasibility aspects in case two instruments have similar quality criteria, which will include considerations such as availability of OMI across multiple languages, length of OMI (in relation to burden of completion for example), and license fees.

### Phase 7: determine “case-mix” factors

Patient characteristics that could differ between countries/healthcare systems and/or could be predictive of outcomes are considered potential candidate case-mix factors. In addition, system level factors will be taken into account. To identify potential case-mix factors for AYAs, all steps from the “what” to measure process described above will be iterated.

A complete overview of the project can be found in Additional file 1: Appendix Table A2.

## Discussion

To our knowledge, there is currently no COS for AYAs available covering the full domain of outcomes. It is hoped that this COS will be adopted as a minimum set of outcomes that should be reported and measured within research and clinical practice for AYAs. The researchers propose a rigorous approach to the development of this COS, which adheres to best practice guidance from the COMET handbook, COS reporting guidelines, and other protocols which have adopted COS methodologies for other health conditions [59]. The COS incorporates the perspectives of multiple stakeholders from research/academic, health professional/policy makers, and patient communities. A well-developed and fully disseminated COS will ensure that relevant outcomes are measured using appropriate and inclusive measurement instruments for AYAs globally. The standardised COS will encourage effective patient monitoring and enhance clinician-patient shared decision-making, benchmarking (within and between hospitals/countries) by providing quality outcome information to providers and institutions to drive transparency and improvement, establish policy, increase research efficiency and global collaboration, improve evidence synthesis, and reduce research waste to accelerate the improvement of outcomes for AYAs.

### Development and implementation challenges

The heterogeneity of AYAs regarding developmental and life stages (adolescence, emerging and young adulthood), the high number of histological subtypes and hence, the broad treatment landscape, different places of care (e.g. paediatric vs. adult vs. AYA units; public vs. private institutions; urban vs. rural; academic vs. non-academic institutions; and availability of an AYA care program at institutions), and diverse healthcare systems as well as social and cultural contexts, make it challenging to develop a single AYA-specific COS that meets the needs of research, clinical practice, policymakers and industry [60]. We therefore propose a flexible strategy with a universally applicable COS for AYAs that captures AYA-specific outcomes that crosscut a majority of cancers connected with disease-specific COSs (e.g., for breast cancer [61]) and target domains that are unique to the cancer and its treatment. Important factors, such as developmental life stage and place of care can be accounted for as case-mix factors in the AYA-specific COS. Up to now, most COS are developed for specific conditions. This means that we have to align with existing initiatives developing COS for specific tumour types (no age restrictions). Therefore, we clearly make a distinction when reviewing outcomes of interest between the ones that are cancer-generic or tumour-specific versus the ones that are AYA age-specific. In case no tumour-specific COS is available when we finalise our COS, we will advise to add the tumour-specific outcomes as identified by us to the COS for AYAs used in research and clinical practice, to make sure no relevant disease-specific outcomes are missed [62].

Another challenge will be effective uptake of the COS by several stakeholders. Within the EU Horizon Europe (HORIZON-HLTH-2021-CARE-05) and UK Research and Innovation (UKRI)—under the UK government’s Horizon Europe funding guarantee [grant number 10038931]—project entitled STRONG-AYA [51], we will work on the implementation of the COS in five national healthcare systems (France, Italy, the Netherlands, United Kingdom and Poland) and establishment of national infrastructures for outcome data management and clinical decision-making and a pan-European ecosystem that also welcomes future (European) countries. In addition, we will disseminate STRONG-AYA COS outcomes and facilitate interactions between national and pan-European stakeholders to develop data-driven analysis tools to process and present relevant outcomes, establish feedback loops for AYAs and the healthcare systems, and improve the reporting and assessment of output towards policy makers.

### Future ambitions

Although all efforts will be made to encourage international participation in the development of the COS, this will be limited by the inability to conduct the study in languages other than English. With the support of an EU Horizon Europe grant and UK Research and Innovation (UKRI) grant we will be able to translate the COS in four other languages (Dutch, Italian, Polish and French). Future funding opportunities should be explored to make the COS available in other languages.

### Supplementary Information

**Additional file 1: Appendix Text A1.** Search string for the literature review per database. **Appendix Table A1. **Stratification matrix. **Appendix Table A2. **Project overview.

## Data Availability

No new data were created or analyzed in this study. Data sharing is not applicable to this article.
