# Supplementary material for: Protocol for the development of a Core Outcome Set (COS) for Adolescents and Young Adults (AYAs) with cancer
Source: BMC Cancer. 2024 Jan 24;24:126. doi: 10.1186/s12885-023-11716-2 (PMC10809623; doi:10.1186/s12885-023-11716-2)
Supplement: Supplementary file 1 — Additional file 1: Appendix Text A1. Search string for the literature review per database. Appendix Table A1. Stratification matrix. Appendix Table A2. Project overview. [file 12885_2023_11716_MOESM1_ESM.docx]

**Appendix**

**Appendix Text A1. Search string for the literature review per database**

**Embase:**

('malignant neoplasm'/mj/exp OR 'neoplasm'/mj/de OR 'oncology'/mj/exp OR 'metastasis'/mj/exp OR 'myelodysplastic syndrome'/mj/de OR 'cancer patient'/mj/exp OR 'digestive system tumor'/mj/exp OR 'cancer mortality'/mj/de OR 'cancer survival'/mj/exp OR 'cancer diagnosis'/mj/exp OR (neoplas* OR cancer* OR malign* OR tumor* OR tumour* OR carcinoma* OR oncolog* OR melanom* OR sarcoma* OR leukemi* OR leukaemi* OR lymphoma* OR glioblastoma* OR metastas* OR myelom* OR hepatoblastoma* OR medulloblastoma* OR glioma* OR myelodysplastic* OR liposarcom* OR osteosarcoma* OR rhabdomyosarcoma* OR adenocarcinoma* OR chordoma* OR aml OR nhl OR craniopharyngioma* OR Hodgkin-disease* OR hepatoma* OR myeloma* OR multiple-myeloma* OR blastoma* OR neuroblastoma* OR ganglioneuroblastoma* OR chondrosarcoma*):ti) AND ('young adult'/mj/de OR 'adolescent'/mj/de OR (juvenil* OR adolescen* OR preadolescen* OR youth* OR young-adult* OR teen* OR puber* OR pubescen* OR highschool* OR AYA OR TYA OR young*-patient*):ti) NOT ([Conference Abstract]/lim OR [Conference Review]/lim) NOT ([animals]/lim NOT [humans]/lim) NOT ('case report'/de OR 'editorial'/de OR (case-report):ti) AND [ENGLISH]/lim

**Medline:**

(exp *Neoplasms/ OR exp *Medical Oncology/ OR exp *Myelodysplastic Syndromes/ OR *Cancer Survivors/ OR *Early Detection of Cancer/ OR (neoplas* OR cancer* OR malign* OR tumor* OR tumour* OR carcinoma* OR oncolog* OR melanom* OR sarcoma* OR leukemi* OR leukaemi* OR lymphoma* OR glioblastoma* OR metastas* OR myelom* OR hepatoblastoma* OR medulloblastoma* OR glioma* OR myelodysplastic* OR liposarcom* OR osteosarcoma* OR rhabdomyosarcoma* OR adenocarcinoma* OR chordoma* OR aml OR nhl OR craniopharyngioma* OR Hodgkin-disease* OR hepatoma* OR myeloma* OR multiple-myeloma* OR blastoma* OR neuroblastoma* OR ganglioneuroblastoma* OR chondrosarcoma*).ti.) AND (*Young Adult/ OR *Adolescent/ OR (juvenil* OR adolescen* OR preadolescen* OR youth* OR young-adult* OR teen* OR puber* OR pubescen* OR highschool* OR AYA OR TYA OR young*-patient*).ti.) NOT (news OR congres* OR abstract* OR book* OR chapter* OR dissertation abstract* OR editorial*).pt. NOT (exp animals/ NOT humans/) NOT (Case Reports/ OR (case-report).ti.) AND english.la.

**Cochrane:**

((neoplas* OR cancer* OR malign* OR tumor* OR tumour* OR carcinoma* OR oncolog* OR melanom* OR sarcoma* OR leukemi* OR leukaemi* OR lymphoma* OR glioblastoma* OR metastas* OR myelom* OR hepatoblastoma* OR medulloblastoma* OR glioma* OR myelodysplastic* OR liposarcom* OR osteosarcoma* OR rhabdomyosarcoma* OR adenocarcinoma* OR chordoma* OR aml OR nhl OR craniopharyngioma* OR Hodgkin-disease* OR hepatoma* OR myeloma* OR multiple-myeloma* OR blastoma* OR neuroblastoma* OR ganglioneuroblastoma* OR chondrosarcoma*):ti) AND ((juvenil* OR adolescen* OR preadolescen* OR youth* OR young-adult* OR teen* OR puber* OR pubescen* OR highschool* OR AYA OR TYA OR young-patient*):ti) NOT "conference abstract":pt

**Web of Science:**

TI=(((neoplas* OR cancer* OR malign* OR tumor* OR tumour* OR carcinoma* OR oncolog* OR melanom* OR sarcoma* OR leukemi* OR leukaemi* OR lymphoma* OR glioblastoma* OR metastas* OR myelom* OR hepatoblastoma* OR medulloblastoma* OR glioma* OR myelodysplastic* OR liposarcom* OR osteosarcoma* OR rhabdomyosarcoma* OR adenocarcinoma* OR chordoma* OR aml OR nhl OR craniopharyngioma* OR Hodgkin-disease* OR hepatoma* OR myeloma* OR multiple-myeloma* OR blastoma* OR neuroblastoma* OR ganglioneuroblastoma* OR chondrosarcoma*)) AND ((juvenil* OR adolescen* OR preadolescen* OR youth* OR young-adult* OR teen* OR puber* OR pubescen* OR highschool* OR AYA OR TYA OR young*-patient*)) NOT ((animal* OR rat OR rats OR mouse OR mice OR murine OR dog OR dogs OR canine OR cat OR cats OR feline OR rabbit OR cow OR cows OR bovine OR rodent* OR sheep OR ovine OR pig OR swine OR porcine OR veterinar* OR chick* OR zebrafish* OR baboon* OR nonhuman* OR primate* OR cattle* OR goose OR geese OR duck OR macaque* OR avian* OR bird* OR fish*) NOT (human* OR patient* OR women OR woman OR men OR man))) NOT DT=(Meeting Abstract OR Meeting Summary) NOT TI=(case-report) NOT DT=(Editorial Material) AND LA=(English)

**Google Scholar:**

AYA|TYA|adolescents|adolescent|"young adult|adults" neoplasm|cancer|malignancy|tumor|tumour|carcinoma|oncology|melanoma|sarcoma|leukemia|leukaemia|lymphoma|glioblastoma|metastasis|myeloma

AYA|TYA|adolescents|adolescent|'young adult|adults' neoplasm|cancer|malignancy|tumor|tumour|carcinoma|oncology|melanoma|sarcoma|leukemia|leukaemia|lymphoma|glioblastoma|metastasis|myeloma

**Appendix Table A1. Stratification matrix**

|  |  | **Adolescents**  **(13-17 years old)**  (25%) | | **Emerging adults**  **(18-25 years old)**  (30%) | | **Young adults**  **(26-39 years old)**  (45%) | | **Overall sample**  (100%) |
| --- | --- | --- | --- | --- | --- | --- | --- | --- |
|  |  | On-treatment | Off-treatment | On-treatment | Off-treatment | On-treatment | Off-treatment |  |
| **Male**  (50%) | **Disease severity**  Low |  |  |  |  |  |  |  |
|  | **Disease severity**  High |  |  |  |  |  |  |  |
| **Female**  (50%) | **Disease severity**  Low |  |  |  |  |  |  |  |
|  | **Disease severity**  High |  |  |  |  |  |  |  |

**Appendix Table A2. Project overview**

| **Level 1** | **Level 2** | **Level 3** | **Level 4** |
| --- | --- | --- | --- |
| Pre-phase | Phase 1: Scope definition |  |  |
|  | Phase 2: Establishment of the need for a COS for AYAs |  |  |
|  | Phase 3: Composition of Working Group |  |  |
| Study protocol development | Phase 4: Study protocol development |  |  |
| Determination of outcomes, outcome measures and case-mix factors | Phase 5: Determine “what” to measure – the outcomes in the COS | Step 1: A literature review | Title/ abstract screening |
|  |  |  | Full text screening |
|  |  |  | Data extraction |
|  |  | Step 2: Identifying and filling the gaps in existing knowledge by interviews and focus groups with stakeholders | Sampling |
|  |  |  | Data collection |
|  |  |  | Data analysis |
|  |  | Step 3: Eliciting views about important outcomes in a Delphi consensus process | Sample and recruitment |
|  |  |  | Round 1 |
|  |  |  | Round 2 |
|  |  |  | Round 3 |
|  |  | Step 4: Consensus meeting |  |
|  |  | Step 5: Dissemination and implementation strategy |  |
|  | Phase 6: Determine “how” to measure the COS | Step 1: Conceptual considerations |  |
|  |  | Step 2a: Identifying existing OMIs |  |
|  |  | Step 2b: Identifying measurement properties of identified OMIs |  |
|  |  | Step 3: Quality assessment of OMIs |  |
|  |  | Step 4: Selection of OMIs for the COS |  |
|  |  | Step 5: Stakeholder’s meeting |  |
|  | Phase 7: Determine “case-mix“ factors | Step 1: A literature review |  |
|  |  | Step 2: Identifying and filling the gaps in existing knowledge by interviews and focus groups with stakeholders |  |
|  |  | Step 3: Eliciting views about important outcomes in a Delphi consensus process |  |
|  |  | Step 4: Consensus meeting |  |
|  |  | Step 5: Dissemination and implementation strategy |  |
